# How great was the influence of his origins and descendants on Charcot's behaviors?

**DOI:** 10.1055/s-0045-1806921

**Published:** 2025-05-09

**Authors:** Hélio A. Ghizoni Teive, Léo Coutinho, Carlos Henrique Ferreira Camargo, Gustavo L. Franklin, Olivier Walusinsi

**Affiliations:** 1 Universidade Federal do Paraná, Hospital de Clínicas, Departamento de Medicina Interna, Serviço de Neurologia, Curitiba PR, Brazil.; 2 Universidade Federal do Paraná, Hospital de Clínicas, Programa de Pós-Graduação em Medicina Interna, Grupo de Doenças Neurodegenerativas, Curitiba PR, Brazil.; 3 Pontifícia Universidade Católica do Paraná, Faculdade de Medicina, Departamento de Medicina Interna, Curitiba PR, Brazil.; 4 Private Practice, Brou, France.

**Keywords:** History of Medicine, Neurology, Genealogy and Heraldry, Jean-Martin Charcot, History, 19th Century

## Abstract

The authors briefly present important data about Professor Charcot's genealogy, discussing possible influences of his family history on his behavior, his personality, and the implications for his brilliant scientific career and professional success. This article adds some data that was ignored in previous biographies.

## INTRODUCTION

**Figure 1:**
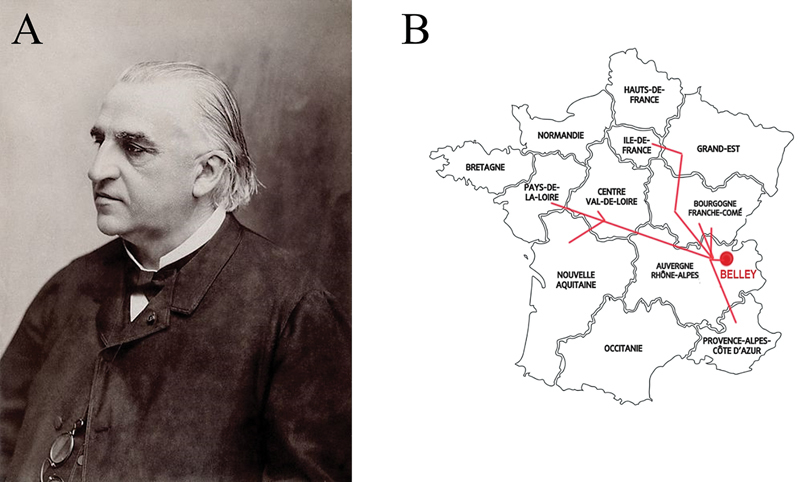
( **A** ) Professor Jean-Martin Charcot (1825–1893). Source: Wikimedia commons. ( **B** ) Charcot family origins. Source: Drawn by the authors (CHFC).

Jean-Martin Charcot ( Figure 1A ), one of the most renowned neurologists of the nineteenth century, is recognized for his contributions in many fields of medicine. 1 2 3 4 5

Several studies 1 2 6 7 8 9 10 have emphasized his achievements, personality, and behavior. 1 2 6 7 8 9 10 Charcot was described as austere and reserved; a man of few words and gestures, with an impenetrable face, authoritarian, skeptical, and sarcastic. 1 2 11 12 13

However, data about his genealogy are scarce. The present paper discusses data on Charcot's genealogy and its influence on his life.

## CHARCOT'S ORIGINS

The origin of the Charcot family is thought to be eastern France. The name is found in Ain (Belley since the beginning of the 17th century), Haute-Savoie and Allier. The oldest known ancestors of Jean-Martin Charcot, Dider and Marguerite Poulot Charcot, lived in Juzennecourt, Haute-Marne, in Champagne-Ardenne, near Germany. They likely descended from one of several families from the currently-called region of Bourgogne-Franche-Comté ( Figure 1B ). 14 15

The meaning of this family name remains uncertain. Genealogists evoke the term *charco* , from the Arpitan Language dialect then spoken in the Jura department, which would have designated a laborer, doing heavy work, or a scapegoat. However, it could also be the old French *charcois* (“carcass”). 15 16

**Figure 2:**
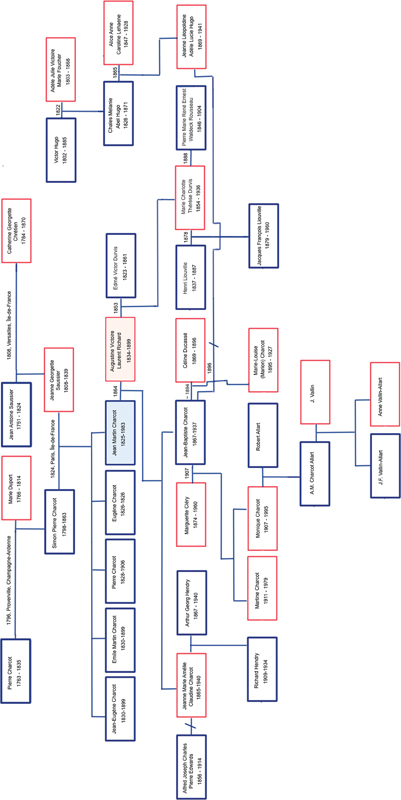
Professor Charcot's genealogy. Source Drawn by the authors (CHFC, GF, and OW). Note: Details of this genealogy can be found in: family beginnings – https://www.familysearch.org/tree/pedigree/landscape/GFM7-343 ; Charcot's first known ancestor – https://www.familysearch.org/tree/pedigree/landscape/GFMM-3BP ; of Jean-Martin Charcot himself – https://www.familysearch.org/tree/pedigree/landscape/GFMM-3BP .

Jean-Martin Charcot was born on November 29, 1825, in Paris, and died in 1893, in Montsauche-les-Settons, in Burgundy. He was the eldest son of Simon-Pierre Charcot (1798–1863), a carriage builder, and Jeanne-Georgette Saussier (1808–1839). 1 2 6 7 8 His siblings were: Eugène Charcot, born in 1826, who died 14 days after birth; Pierre-Martin Charcot, born in 1828, who succeeded his father as carriage builder and died in 1906; Émile-Martin Charcot, born in 1830, an officer in the army who died in 1899; and Jean-Eugène Charcot, born in 1831, a non-commissioned officer in the African Army who disappeared in Senegal on July 8, 1869, at the age of 37 ( Figure 2 ). 1 2 6 7 8

Charcot's mother, Jeanne, was the daughter of Jean Antoine Saussier, a locksmith turned carriage builder, whose mother was of the Guérard family, and of Catherine Georgette Chrétien. 1 2 6 7 8 His father, Simon-Pierre, was the son of Pierre Charcot (1763–1835) and Marie Duport (1766–1814). 14

## JEAN-MARTIN CHARCOT AND AUGUSTINE-VICTOIRE LAURENT RICHARD

Jean-Martin Charcot got married in 1864, at the age of 38, to Augustine-Victoire Laurent (1834–1899), who was 29 years old. Augustine-Victoire was the daughter of Charles Vincent Claude Laurent, the Parisian clothier and director of the Maison Laurent-Richard, and Augustine Victoire Charlotte Richard. She was first married to Edmé Victor Durvis (1823–1861), but after 8 years of marriage, Edmé Durvis died at the age of 38. The couple had one daughter, Marie Charlotte Thérèse Durvis (1854–1936), whom Charcot helped raise. Augustine-Victoire was widowed for 3 years before marrying Professor Charcot. 6 7 8 14

Marie Charlotte Thérèse Durvis was married at the age of 24 to Henri Liouville (1837–1887) and had a son named Jacques-François Liouville (1879–1960), but Henri died prematurely. In 1888, at age 34, Marie Charlotte married Pierre Marie René Ernest Waldeck Rousseau (1846–1904), an important lawyer and politician. The couple had no children. 1 2 6 7 8 17

## CHARCOT DESCENDANTS

Charcot and Augustine-Victoire had a couple of children: Jeanne Marie Amélie Claudine Charcot, born in 1865, who died in 1940, at the age of 75, and Jean-Baptiste Charcot, born in 1867, who died in 1937, at the age of 70. 1 2 6 7 8

Jeanne married Alfred Joseph Charles Pierre Edwards (1856–1914) and divorced soon after. Her second marriage was to Arthur Georg Hendry (1867–1940). Their son, Richard Hendry, died of tuberculosis at the age of 25, in 1934. 1 2 6 7 8

Jean-Baptiste initiated a medical career, training in neurology at Hôpital de la Salpêtrière under the supervision of his father and his successors, but later abandoned it, becoming a famous maritime explorer. 6 7 8 18 19

Jean-Baptiste Charcot had a relationship with Céline Ducassé (1869–1896), having a daughter named Marie-Louise (Marion) Charcot (1895–1927). In 1896, he married Léopoldine Clémence Adèle Lucie “Jeanne” Hugo (1869–1941), granddaughter of the writer Victor Hugo. She was previously married to Léon Daudet (1867–1942), son of the writers Alphonse Daudet (1840–1897) and Julia Daudet, divorcing in 1895. Jean-Baptiste Charcot and Jeanne Hugo Charcot divorced in 1905, after Jean-Baptiste Charcot's trip to Antarctica, on the grounds of desertion. 18 19

After that, Jean-Baptiste married Marguerite Cléry (1874–1960) in 1907. They had 2 daughters, Martine Charcot (1911–1979), who had no children, and Monique Charcot (1907–1995), who married Robert Allart. 1 2 18 19 The couple had 1 daughter, Anne-Marie Allart Charcot (1936–2017), who married J. Vallin, producing 2 children, J.F. Vallin-Charcot and Anne Vallin-Charcot. 2

## PROFESSOR JEAN-MARTIN CHARCOT'S SCIENTIFIC CAREER AND HIS RELATIONSHIP WITH HIS FAMILY

Professor Charcot's brilliant career appears to have been unaffected by his troubled family life. 1 2 His mother, Jeanne Saussier, died at the age of 30, when Charcot was 14 years old; among his brothers, Eugène died shortly after his birth, and Jean-Eugène disappeared in Senegal in 1869, at the age of 37. 1 2 3 4 5 6 7 8 13 These facts may provide insights into Professor Charcot's behavior and personality. 6 7 8 11 12 13

Charcot was already 38 years old when he married a wealthy widow, Augustine-Victoire. This provided him a quick social ascent, as he came from a modest family. The fame that Charcot acquired in the second half of the nineteenth century granted him an illustrious clientele and financial stability. 1 2 13 It is estimated that a consultation with Professor Charcot, at the pinnacle of his fame, would cost around 160 dollars. The mansion he acquired at 217 Boulevard Saint Germain, known as the *Hôtel de Varengeville* , had an estimated value of 1.05 million French francs. The couple also inherited from Augustine-Victoire's parents a country house in the town of Neuilly-sur-Seine. 2

As was the norm in most families in nineteenth-century France, Charcot's family was centered on paternal authority, as exemplified by the fact that Jean-Baptiste Charcot pursued his medical career to honor his father's wishes. 18 19 After Charcot's death in 1893, the disbursement of the family fortune progressively occurred. 1 2 13 His children were married several times, and with his son, Jean-Baptiste Charcot, abandoning his medical career and due to his expenses with maritime expeditions, the Charcot family lost much of its income and prestige. 1 2 13 18 19

Charcot and many of his descendants presented significant longevity, given that life expectancy in Europe throughout the nineteenth century varied from 33.3 to 42.7 years. 20

In conclusion, an analysis of Charcot's genealogy enables us to better understand his personality and trajectory. His simple and troubled origin may have contributed to his personality, but it did not interrupt his social and academic rise.

## References

[1] Guillain G J-M Charcot; his life-his work New York Paul B. Hoeber 1959 3 66

[2] Goetz C G Bonduelle M Gelfand T Charcot: Constructing Neurology New York Oxford Univ Press 1995 149 151

[3] Bogousslavsky J Boller F Jean-Martin Charcot and art: relationship of the “founder of neurology” with various aspects of art Prog Brain Res 2013 203 185 199 10.1016/B978-0-444-62730-8.00007-4 24041281

[4] Goetz C G Jean-Martin Charcot (1825–1893) J Neurol 2005 252 374 375 10.1007/s00415-005-0776-1

[5] Kumar D R Aslinia F Yale S H Mazza J J Jean-Martin Charcot: the father of neurology Clin Med Res 2011 9 01 46 49 10.3121/cmr.2009.883 20739583 PMC3064755

[6] Guinon G Charcot Intime. Hommage à Charcot Paris Med (Paris) 1925 511 516

[7] Souques A Charcot Intime Presse Med 1925 42 693 698

[8] Bonduelle M [The intimate Charcot] Rev Neurol (Paris) 1994 150(8-9):524 528 7754287

[9] Lellouch A [Charcot, discoverer of diseases] Rev Neurol (Paris) 1994 150(8-9):506 510 7754284

[10] Teive H AG Munhoz R P Barbosa E R Little-known scientific contributions of J-M Charcot Clinics (Sao Paulo) 2007 62 03 211 214 10.1590/s1807-59322007000300003 17589659

[11] Teive H AG Germiniani F MB Munhoz R P Charcot's irony and sarcasm Arq Neuropsiquiatr 2017 75 06 402 404 10.1590/0004-282x20170062 28658411

[12] Bonduelle M [Charcot. Dates. Legend and reality] Hist Sci Med 1994 28 04 289 295 11640481

[13] Poirier J Jean-Martin Charcot (1825–1893): sa personne, sa personnalité, son personnageNeurologie Libérale; 03: Juillet-août-septembre, 2013

[14] FamilySearch Charcot – Belley - Steam (Pedigree – Landscape). 1[https://www.familysearch.org/tree/pedigree/landscape/GFM7-343], accessed on August 13, 2022

[15] Geneanet Origine du nom CHARCOT[https://www.geneanet.org/nom-de-famille/CHARCOT], accessed on August 13, 2022

[16] Anonymous Noms de famille – Noms commençant par C[https://web.archive.org/web/20220204225112/http://jeantosti.com/noms/c5.htm], accessed on August 13, 2022

[17] Surhone L M Timpledon M T Marseken S F Pierre Waldeck-Rousseau Betascript Publishers, Mauritius 2010

[18] Teive H AG Germiniani F MB Camargo C HF Broken dynasty: how Jean Batiste Charcot relinquished his father's neurological empire to conquer the seven seas Neurol Sci 2018 39 04 765 768 10.1007/s10072-018-3258-9 29383615

[19] Teive H A Munhoz R P Simões J C Charcot's son, commander Jean-Baptiste Charcot: from neurology to “Pourquoi Pas?” Arq Neuropsiquiatr 2012 70 04 305 307 10.1590/s0004-282x2012000400016 22510742

[20] UN WPP (2022), HMD (2023), Zijdeman et al. (2015), Riley (2005) – with minor processing by Our World in Data“Life expectancy at birth – Various sources – period tables” [dataset]. Human Mortality Database, “Human Mortality Database”; United Nations, “World Population Prospects 2022”; United Nations, “World Population Prospects”; Zijdeman et al., “Life Expectancy at birth 2”; James C. Riley, “Estimates of Regional and Global Life Expectancy, 1800-2001” [original data]

