# Erratum

**DOI:** 10.1055/s-0045-1809365

**Published:** 2025-05-20

In the article “How great was the influence of his origins and descendants on Charcot's behaviors?”, published in the Arquivos de Neuro-Psiquiatria 2025;83(3):s0045-1806921, under DOI 10.1055/s-0045-1806921 .

Where it reads:

Olivier Walusinsi

It should be:

Olivier Walusinski

